# Cell-intrinsic sphingosine kinase 2 promotes macrophage polarization and renal inflammation in response to unilateral ureteral obstruction

**DOI:** 10.1371/journal.pone.0194053

**Published:** 2018-03-08

**Authors:** Mallika Ghosh, Shobha Thangada, Oisharya Dasgupta, Kamal M. Khanna, Harold T. Yamase, Michael Kashgarian, Timothy Hla, Linda H. Shapiro, Fernando A. Ferrer

**Affiliations:** 1 Center for Vascular Biology, University of Connecticut School of Medicine, Farmington, CT, United States of America; 2 Department of Cell Biology, University of Connecticut School of Medicine, Farmington, CT, United States of America; 3 Department of Immunology, University of Connecticut School of Medicine, Farmington, CT, United States of America; 4 Department of Pathology, University of Connecticut School of Medicine, Farmington, CT, United States of America; 5 Department of Pathology, Yale University Cancer Research Center, New Haven, CT, United States of America; 6 Vascular Biology Program, Boston Children's Hospital, Boston, MA, United States of America; 7 Section of Pediatric Urology, Children's Hospital of Omaha, Department of Surgery, University of Nebraska School of Medicine, Omaha, NE, United States of America; UCL Institute of Child Health, UNITED KINGDOM

## Abstract

Sphingosine Kinase-2 (Sphk2) is responsible for the production of the bioactive lipid Sphingosine-1 Phosphate, a key regulator of tissue repair. Here we address the *in vivo* significance of Sphingosine Kinase -2 in renal inflammation/fibrosis in response to unilateral ureteral obstruction using both genetic and pharmacological strategies. Obstructed kidneys of *Sphk2*^*-/-*^ mice showed reduced renal damage and diminished levels of the renal injury markers TGF**β**_1_ and **α**SMA when compared to wild type controls. We found a consistently significant increase in anti-inflammatory (M2) macrophages in obstructed *Sphk2*^*-/-*^ kidneys by flow cytometry and a decrease in mRNA levels of the inflammatory cytokines, MCP1, TNF**α**, CXCL1 and IL**β**_1_, suggesting an anti-inflammatory bias in the absence of Sphk2. Indeed, metabolic profiling showed that the pro-inflammatory glycolytic pathway is largely inactive in *Sphk2*^*-/-*^ bone marrow-derived macrophages. Furthermore, treatment with the M2-promoting cytokines IL-4 or IL-13 demonstrated that macrophages lacking Sphk2 polarized more efficiently to the M2 phenotype than wild type cells. Bone marrow transplant studies indicated that expression of Sphk2^-/-^ on either the hematopoietic or parenchymal cells did not fully rescue the pro-healing phenotype, confirming that both infiltrating M2-macrophages and the kidney microenvironment contribute to the damaging Sphk2 effects. Importantly, obstructed kidneys from mice treated with an Sphk2 inhibitor recapitulated findings in the genetic model. These results demonstrate that reducing Sphk2 activity by genetic or pharmacological manipulation markedly decreases inflammatory and fibrotic responses to obstruction, resulting in diminished renal injury and supporting Sphk2 as a novel driver of the pro-inflammatory macrophage phenotype.

## Introduction

The bioactive lipid Sphingosine-1-Phosphate (S1P) and its five receptors S1PR_1-5_ play important roles in human health and disease. S1P has been demonstrated to participate in the regulation of numerous cellular processes such as cytoskeletal rearrangement, cell migration, angiogenesis, vascular maturation, apoptosis, inflammation and immune cell trafficking[1,2]. The majority of S1P is produced as the result of the hydrolysis of sphingomyelin to sphingosine, which is then phosphorylated to S1P by either of two closely related sphingosine kinases, Sphk1 or Sphk2. Mice harboring a combined deletion of the two kinases die in utero, while mice with a single isoform deletion are viable. Both isoforms are significantly active at baseline and it is thought that Sphk1 is primarily responsible for maintaining intracellular and circulating S1P levels. While the contribution of Sphk2 is less clear in this regard, Sphk1 activity and consequent plasma S1P levels are significantly increased in *Sphk2*^*-/-*^ mice[3], suggesting that the Sphk2 isoform also contributes to systemic S1P levels. Significant structural homology exists between the two isoforms, but their clearly differential cellular localization and sometimes opposing functions[4,5] may also suggest distinctly different roles. While Sphk1 has historically been more intensely studied due to its prominent role in tumor cell proliferation, recent investigations have increasingly focused on understanding potentially unique functions of Sphk2.

Obstructive renal injury is the most common cause of chronic kidney disease in children and similar to ischemia reperfusion injury, when damage to the obstructed kidney occurs it is accompanied by deregulation of the immune and vascular systems. The sphingolipid signaling pathways are key modulators of various inflammatory processes[6]. Given this information, in the present study we specifically investigated the contribution of Sphk2 to inflammation-induced renal damage resulting from obstructive nephropathy utilizing the Unilateral Ureteral Obstruction (UUO) model from both genetic and pharmacological perspectives. Global *Sphk2*-deficient mice were significantly protected from renal inflammation, thus diminishing the ensuing injury and fibrosis. This phenomenon is recapitulated in wild type mice treated with a specific Sphk2 inhibitor (SK2i), SLP 120701[7] in agreement with recent studies showing similar reductions in renal fibrosis upon Sphk2 lack or inhibition [8,9]. However, these studies did not explore a potential inflammatory basis for these effects. In this regard, our bone marrow transplant experiments indicated that both hematopoietic and parenchymal cells contribute to the deleterious effects of Sphk2 in obstructive injury. Further investigation demonstrated that either deletion or inhibition of Sphk2 protects the kidney from injury in large part by promoting macrophage polarization to the M2 phenotype and identifies Sphk2 as a mediator of macrophage specification and a critical regulator of inflammatory injury. Furthermore, we propose that pharmacologic inhibition of Sphk2 results in a defined, predictable and favorable outcome and that Sphk2 is a viable therapeutic target in the treatment of obstructive renal injury and potentially, unresolved inflammation of any etiology.

## Materials and methods

### Mice

The experiments were performed on 6–8 week old male C57/B6 (wild type) and *Sphk2*^*-/-*^ mice. All animals were given humane care using protocols approved by the institutional Animal Care Committee.

### Euthanasia

Mice were sacrificed by CO2 narcosis followed by cervical dislocation.

### Sphk2 inhibitor (SK2i, SLP 120701) model

Male, 6–8 weeks old C57BL/6 WT mice were treated with SK2i inhibitor (3mg/kg) or vehicle control, daily by i.p three days prior to and following the UUO surgery (6 days total). SK2i **(**SLP 120701, formerly SKX002411) was a kind gift from SphynKx Therapeutics, Charlottesville, VA[7]. Control group received 2% Cyclodextrin (sigma) in which the drug was prepared.

### UUO model

The general procedure of the UUO model has been well described[10]. Briefly, 6–8 weeks old WT and *Sphk2*^*-/-*^ mice were anesthetized using Isofluorane gas and complete ureteral obstruction was performed by ligating left ureter through a posterior flank incision. In the preliminary studies, we have noticed that at 24 hours of UUO, glomerular and tubulointerstitial morphologies were intact and by 72 hours of post-obstruction, renal damage was obvious and limited to tubular atrophy and widened interstitial spaces accompanied by inflammatory cell infiltration. In the present study, mice (n = 6) were sacrificed at 1,3,5 and 7 days (for the genetic model) and 3 and 7 days (for inhibitor model), after the obstruction surgery. The contralateral kidney served as an internal control, is comparable to sham operated kidney.

### Renal pathology and renal injury scoring

Renal tubulointerstitial injury evaluated semi-quantitatively by a renal pathologist in WT, *Sphk2*^*-/-*^, SK2i and vehicle treated mice using H & E and PAS stained slides. Briefly, multiple tubulointerstitial fields were randomly selected and evaluated for tubulointerstitial injury based on tubular dilatation, tubular cell vascularization, tubular atrophy, proximal tubule brush border, cortical thickening, interstitial infiltrates, interstitial edema and interstitial fibrosis as described before [11] on a scale of 0–5 (0- least injury and 5—extensive injury).

### Immunohistochemistry (IHC)

Hematoxylin and eosin, Masson’s trichrome, TGF**β** and **α**-SMA staining performed on 5-μm thick paraffin sections of control and obstructed kidney as described earlier [12]. Ten consecutive fields were examined using Zeiss bright field microscope (20X objective).

### Western blot

Proteins extracted in RIPA buffer from kidney tissues were used for western blotting for TGF**β**1 (Santa Cruz), **α**-SMA and Sphk2 (Abcam) as described earlier [12].

In vitro differentiation of Bone marrow derived macrophages. Bone marrow cells isolated from WT and *Sphk2*^*-/-*^ mice were differentiated into macrophages, in medium containing Macrophage colony stimulating growth factor (20ng/ml-MCSF) [13] and stimulated either with IL4 or IL13 to further differentiate into M2 phenotype [14].

### Flow cytometry

Kidney tissues were harvested and digested with Collagenase1 (Gibco) (50μg/ml), to isolate single cells. 2X10^6^ viable cells were stained with specific markers to exclude T and B Lymphocytes to analyze M2 (CD11b^+^, F4/80^+^, CD206^+^) in CD45^+^ cells [15–17].

### RNA isolation and qRT-PCR analysis

Total RNA was extracted from non-obstructed and obstructed kidneys of WT, *Sphk2*^*-/-*^ and SK2i treated mice and real time PCR was performed as described earlier [12]. The relative fold increase in mRNA expression was normalized to GAPDH and analyzed using RQ manager and Data Assist software.

### Bone marrow transplant experiments

For bone marrow transplant (BMT) experiments recipient wild type mice (CD45.2, 8–10 weeks) were subjected to whole body irradiation from a [^137^ Cs] radiation source (Gamma cell-40) with a total dose of 10Gy for 10 minutes and were treated with neomycin in the drinking water. Donor bone marrow cells were prepared from 6–8 week old WT and *Sphk2*^*-/-*^ mice (CD45.2) and 5X10^6^ cells were injected per mouse via the lateral tail vein immediately after the whole body irradiation of recipient mice. Engraftments were confirmed by flow cytometry analysis of circulating leukocytes pre- and post-transplant. Bone marrow transplanted chimeric mice were allowed to recover for 8 weeks before UUO. Kidneys were harvested at 7days after surgery.

### Cellular bioenergetics-seahorse analysis

As described previously [18] bone marrow derived macrophages from WT and *Sphk2*^*-/-*^ were seeded into each well (80,000 cells/well) of Seahorse Bioscience (Billerica, MA) tissue culture plate and incubated overnight at 37°C. Before analysis cells were washed twice with glucose free DMEM assay medium supplemented with glutamine and incubated for 1 hour at 37°C without CO2. Measurements of glycolytic rate and glycolytic capacity were determined by recording extracellular acidification rate (ECAR, milli pH/min) on a Seahorse Bioscience XF96 Extracellular Flux Analyzer. The injection of glucose was used to measure glycolytic rate (final concentration 25mM), the injection of oligomycin (final concentration 2.5 μM) was used to measure glycolytic capacity and injection of 2-deoxyglucose (2DG) was used to blunt glycolysis (final concentration 20mM). Background from cell free wells was subtracted. N = 6 replicates per treatment and experiment was repeated 2 times.

### S1P measurement from plasma samples

Plasma samples were prepared from WT, *Sphk2*^*-/-*^ and SK2i treated mice for S1P measurement. Samples for S1P measurement were submitted to Lipidomic Core Mass Spectrometry Lab, Medical University of South Carolina, Charleston, South Carolina. Analysis was performed by Liquid Chromatography- ESI Mass Spectrometry (LC-MS) using triple quadrupole mass spectrometer coupled to a Shimadzu LC-20AD LC system as described earlier [3].

### Statistical analysis

All experiments were conducted at least two times using 6 animals in each group. Data presented in graphs represent the means ± SEM for each group. Comparison of 2 groups was performed using an unpaired, 2 tailed t test. Statistical analyses were performed using GraphPad Prism (GraphPad Software Inc.). P values, *P<0.05, ** P<0.01 and *** P<0.001 are considered as statistically significant.

## Results

### Obstruction-induced renal injury is diminished in *Sphk2*^*-/-*^ mice

**Fig 1:**
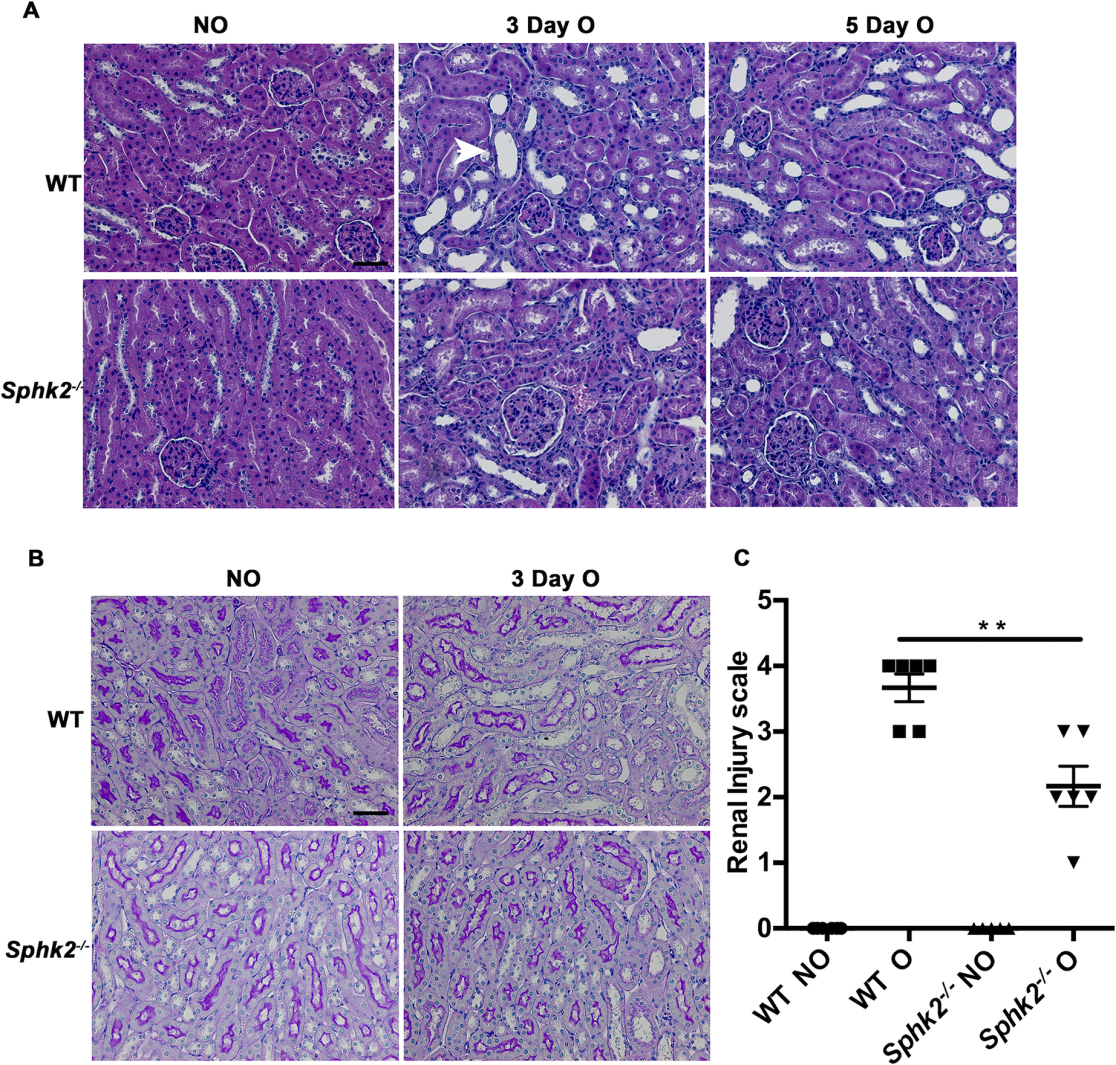
Diminished renal injury in *Sphk2*^*-/-*^ mice after UUO. (A) Paraffin sections were stained with Hematoxylin & Eosin (H&E) and Periodic Acid Schiff (PAS). Representative photomicrographs taken with Zeiss microscope using 40x objective, WT and *Sphk2*^*-/-*^ mice (N = 6) non-obstructed (NO) and obstruced (O) kidneys at day 3 and 5. H&E (A), PAS (B). Infiltration of inflammatory cells, tubular atrophy and widening of interstitial spaces (by H&E stain), and tubular damage (by PAS stain) were considered as the criteria to assess the renal injury. (C) PAS stained slides were blinded and scored for renal injury. A scale of 0–5, zero being the least and 5 being the maximum injury, revealed that *Sphk2*^*-/-*^ mice had diminished renal injury after 3 days of obstruction compared WT (**p<0.01). Experiments were repeated at least two times with N = 6 each time. Original magnification; 20x objective. Scale bar; 50μm.

In the kidney both Sphk1 and 2 are expressed throughout development, predominantly in the metanephric mesenchyme where expression levels increase through maturity. *Sphk2*^*-/-*^ mice are phenotypically normal and we observed no remarkable differences in baseline renal histology by light or electron microscopy between wild type and *Sphk2*^*-/-*^ mice. Flow cytometry studies also revealed that basal immune cell profiles of bone marrow cells were similar between the genotypes, ensuring that any differences observed going forward are not likely due to developmental defects in the absence of Sphk2 (S1A Fig). Permanent unilateral ureteral obstruction was performed on WT and *Sphk2*^*-/-*^ mice and both kidneys were harvested at the peak of the early and late inflammatory responses, 3 and 5 days post-surgery. Evaluation of Sphk2 expression post obstruction revealed an increase in Sphk2 protein levels in the obstructed kidney compared to non-obstructed control (S1B Fig). Hematoxylin and Eosin or Periodic acid–Schiff stained renal sections (Fig 1A and 1B) were evaluated by two independent experienced renal pathologists in a blinded manner. Assessment of renal injury was based on the degree of interstitial infiltration, cortical thickening, tubular dilatation and proximal tubule brush border integrity at day 3 on a scale of increasing damage from 0–5 based on previously published studies[11,19–21] (Fig 1C). Obstructed kidneys of *Sphk2*^*-/-*^ mice exhibited less structural alteration when compared to WT mice as evidenced by reduced tubular dilation, epithelial cell sloughing and basement membrane thickening. There were no remarkable histological changes observed in either non-obstructed or sham operated kidneys, suggesting that Sphk2 deficiency protects kidneys from tissue injury resulting from UUO.

### TGFβ_1_ expression levels are diminished in *Sphk2*^*-/-*^ mice

**Fig 2:**
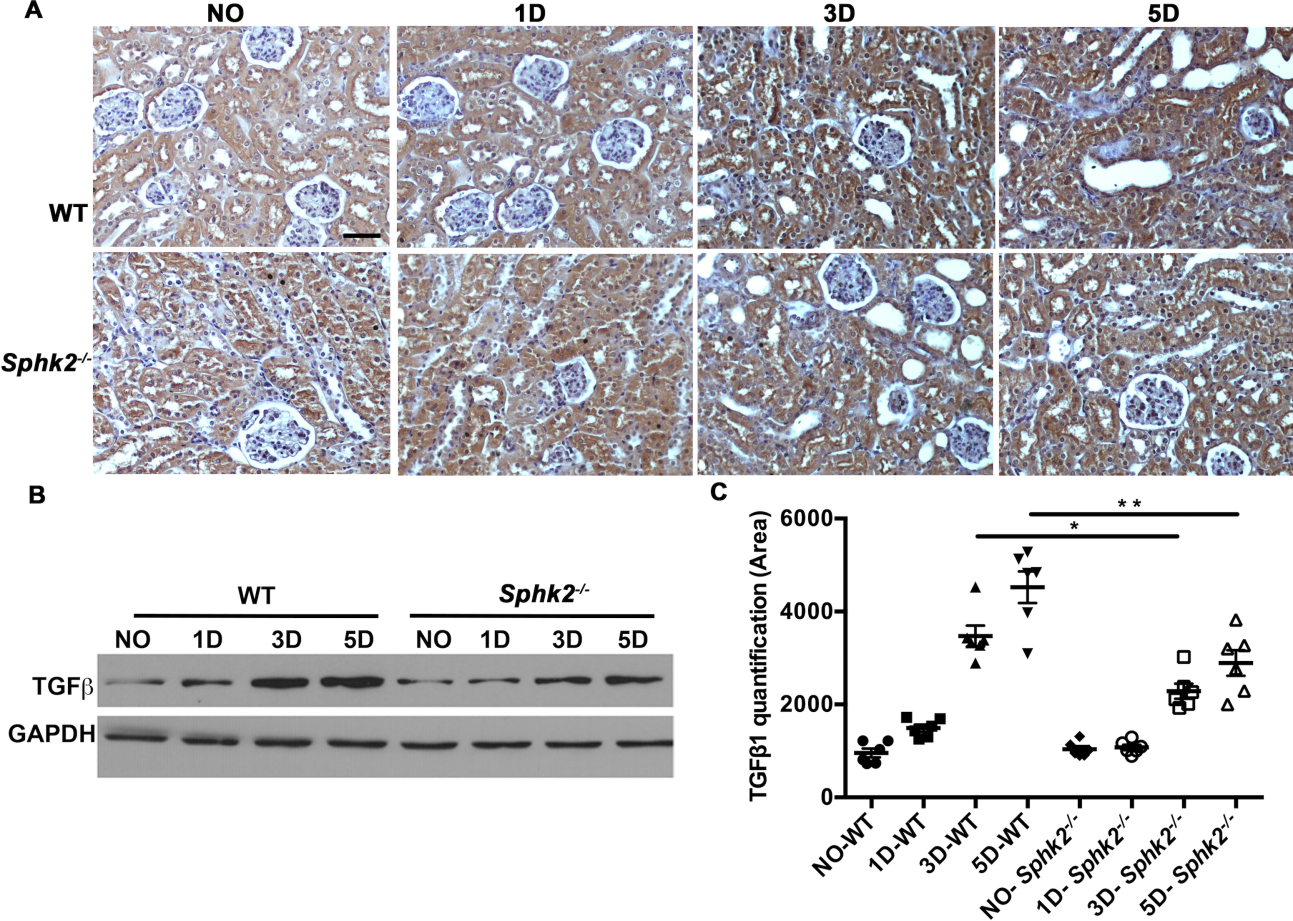
TGFβ_1_ expression is diminished in *Sphk2*^*-/-*^ mice compared to WT. TGF**β**_1_ expression significantly increased in obstructed kidneys than in non-obstructed kidneys (N = 6). (A) Representative photomicrographs show immunohistochemical staining for TGF**β**_1_ in WT mice (top panel) and *Sphk2*^*-/-*^ (bottom panel) at 3 & 5 days after UUO in non-obstructed (NO) and obstructed (O) kidneys. (B, C) Western blot analysis of TGF**β**_1_ in WT and *Sphk2*^*-/-*^ mice at 1, 3 & 5 days and protein bands quantified using Image J and normalized to GAPDH. Results from contralateral non-obstructed kidney were comparable to sham operated kidneys. Experiments were repeated at least two times with N = 6 each time. WT vs *Sphk2*^*-/-*^ at 3 day (*p<0.05); WT vs *Sphk2*^*-/-*^ at 5 day post ligation (**p<0.01). Original magnification; 20x objective. Scale bar; 50μm.

Ureteral obstruction results in increased urinary pressure in the renal tubules leading to tubular dilation and damage. Tissue injury promotes the activation of resident macrophages, progression to a pro-fibrotic phenotype, increased deposition of extracellular matrix proteins, a robust inflammatory response and ultimately, progressive renal disease. We initially assessed protein levels of pro-fibrotic TGF-**β**_1_ as a hallmark of this fibrotic progression [22,23] and found that indeed, tubulointerstitial expression of TGF-**β**_1_ progressively increased in obstructed kidneys from day 3 to day 5 post-obstruction in animals of both genotypes. However, this increase was significantly greater in WT mice when compared to *Sphk2*^***-/-***^ mice as confirmed by IHC and western blot analysis (Fig 2A–2C).

### αSmooth muscle actin levels are reduced in *Sphk2*^*-/-*^ mice

**Fig 3:**
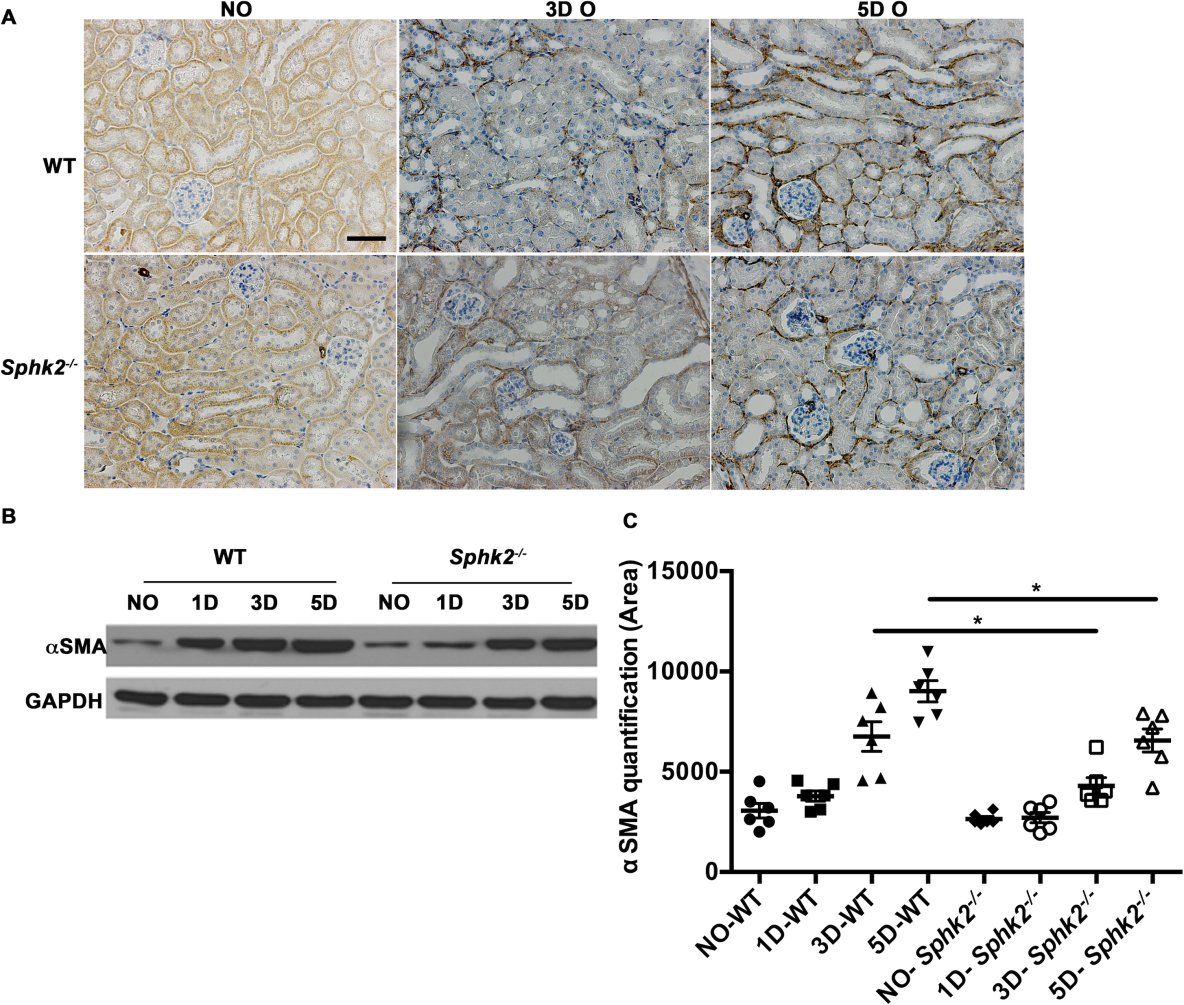
αSmooth muscle actin levels are elevated in WT mice when compared to *Sphk2*^*-/-*^ mice. **(**A) Representative photomicrographs show immunohistochemical staining in non-obstructed (NO) and obstructed (O) kidneys of WT and *Sphk2*^*-/-*^ mice. (B, C) Western blot analysis for **α**SMA in WT and *Sphk2*^-/-^ mice at 1,3 & 5 days after UUO and quantification of protein bands using image J and normalized to GAPDH. Experiments were repeated at least two times with N = 6 each time. WT vs *Sphk2*^*-/-*^ at 3 day (*p<0.05); WT vs *Sphk2*^*-/-*^ at 5 day post ligation (*p<0.05). Original magnification; 20x objective. Scale bar; 50μm.

TGF**β**1 promotes the pathogenesis of renal interstitial fibrosis [24] and is characterized by d*e novo* expression of **α**-SMA. Immunohistochemical staining for **α**-SMA demonstrated that the expression of interstitial **α**-SMA gradually increased over time in obstructed kidneys from both groups, but expression was substantially higher in WT as compared to *Sphk2*^*-/-*^ mice. Similar results were obtained by western blot analysis of protein levels of **α**-SMA (Fig 3A–3C) in whole kidney lysates, supporting reduced myofibroblast transition and disease progression in the absence of Sphk2 following UUO.

### Macrophage profiles are altered in obstructed kidneys of *Sphk2*^*-/-*^ mice

**Fig 4:**
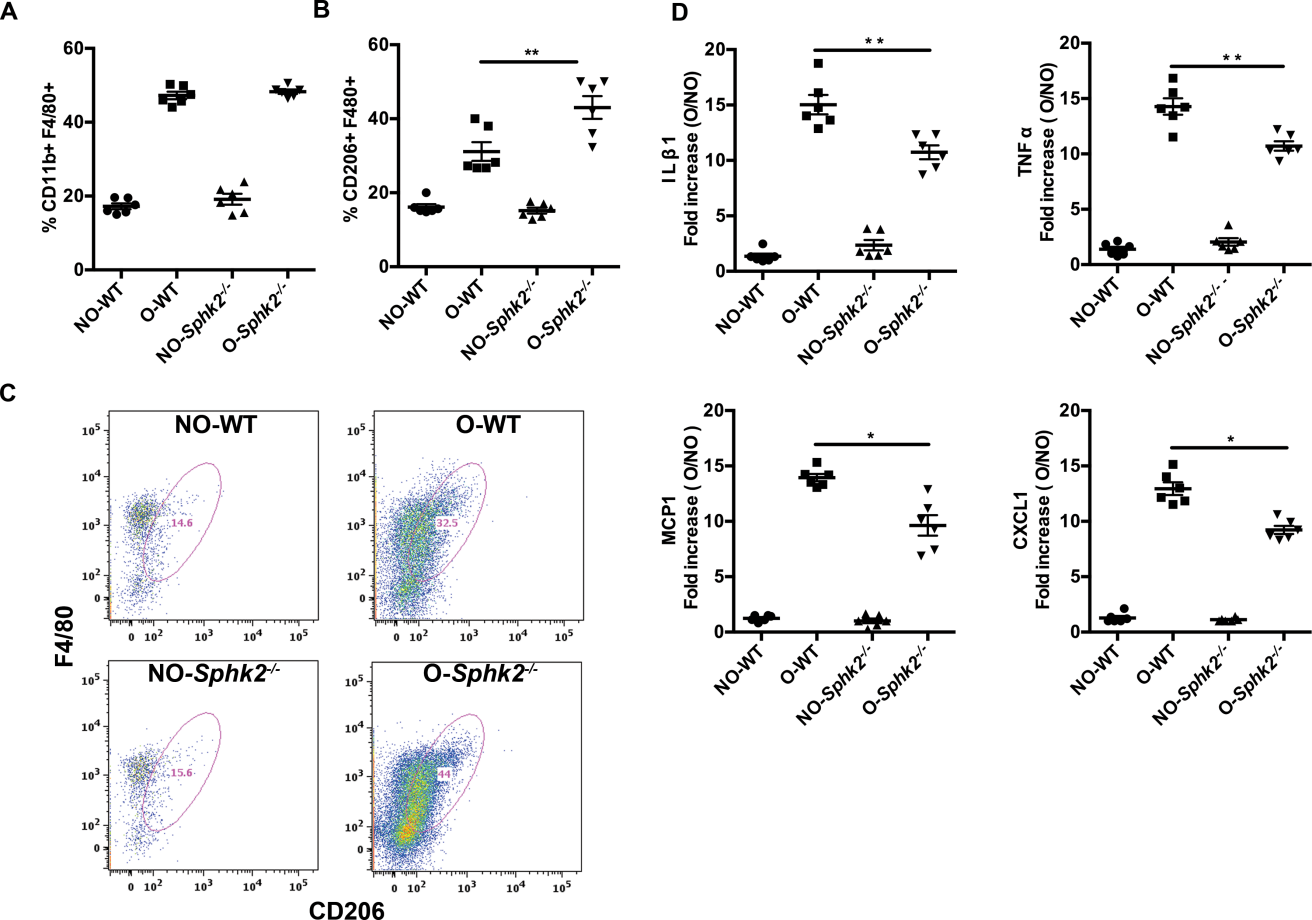
Differential recruitment of macrophage subtypes to kidneys after UUO: M2 phenotype of macrophages population is predominant in obstructed kidneys of *Sphk2*^*-/-*^ mice. (A) Kidney cells from non-obstructed (NO) and obstructed (O) kidneys of WT and *Sphk2*^*-/-*^ mice were isolated by collagenase-1 digestion and stained with other immune cell and macrophage markers for flow cytometry. Total macrophages as identified by CD11b^+^ F4/80^+^ (A) increased after obstruction in both WT and *Sphk2*^*-/-*^ mice, however the specific population of CD11b^+^ F4/80^+^ CD206^+^ macrophages (B) was significantly increased (p<0.05, WT vs. *Sphk2*^*-/-*^ obstructed kidneys) in *Sphk2*^*-/-*^ mice compared to WT mice. (C) Representative pseudo color plots in WT and *Sphk2*^*-/-*^ mice. (*p<0.05). (Data represents average of 3 experiments, n = 6/experiment). **(D)** Total RNA was prepared from contralateral non-obstructed (NO) and obstructed (O) kidneys of WT and *Sphk2*^*-/-*^ mice. Quantitative real time PCR was performed using Fast SYBR Green Master Mix. The relative fold increase in mRNA expression was normalized to GAPDH and fold increase is represented in the graph. QRT PCR data revealed that *Sphk2* mice significantly decreased proinflammatory cytokine mRNA expression levels compared to vehicle treated mice IL1**β**, TNF**α**, MCP-1 and CXCL1. (*p<0.05, WT vs. *Sphk2*^*-/-*^ obstructed kidneys), n = 6. Experiments were repeated at least two times with n = 6 each time.

Tissue damage resulting from ureteral obstruction signals a potent inflammatory response initiated by heterogeneous subsets of both tissue-resident immune cells and circulating inflammatory cells infiltrating at the site of injury. Phenotypically distinct populations of macrophages cooperate to initially clear the wound of dead cell debris and to orchestrate the ongoing inflammatory response (pro-inflammatory or M1 macrophages) and a second group that serves to promote healing, revascularization and resolve inflammation (pro-healing or M2 macrophages). The correct balance of these subpopulations and their expressed cytokines is critical to wound healing and resolution. We tracked potential effects of lack of Sphk2 on the profiles of kidney-resident macrophages from WT and *Sphk2*^*-/-*^ mice subjected to UUO for 3 days by flow cytometry (S3A Fig). Infiltrating macrophages at day 3 of obstruction in both WT and *Sphk2*^*-/-*^ mice were increased in obstructed kidneys compared to non-obstructed and sham kidneys (Fig 4A, CD11b^+^/F4/80^+^/CD11c^—^ negatively selected for CD11c+ dendritic cells). Further analysis illustrated that the macrophage population in *Sphk2*^*-/-*^ obstructed kidneys showed a consistent 10–15% increase in cells expressing high levels of CD206, a marker of the pro-healing M2 macrophage phenotype (CD11b^+^ F4/80^+^ CD206^+^, Fig 4B and 4C). These results indicate that the reduction in renal injury and reduced proinflammatory cytokine levels we observe in the absence of Sphk2 may be due to a bias toward M2 macrophage polarization.

Skewed macrophage profiles would be expected to result in altered cytokine profiles in WT vs *Sphk2*^*-/-*^ obstructed kidneys and contribute to differences in healing. qRT-PCR of total kidney RNA isolated at 3 day post-obstruction indicated that expression levels of the proinflammatory cytokines, IL-1**β** and TNF**α** were significantly lower in *Sphk2*^*-/-*^ kidneys as compared to the WT group (Fig 4D). These results suggest that Sphk2 contributes to inflammatory processes following UUO, perhaps at the level of macrophage polarization or subset infiltration to promote increased injury and a pro-inflammatory environment.

### Bone marrow-derived macrophages from *Sphk2*^*-/-*^ mice are preferentially polarized toward the M2 phenotype

**Fig 5:**
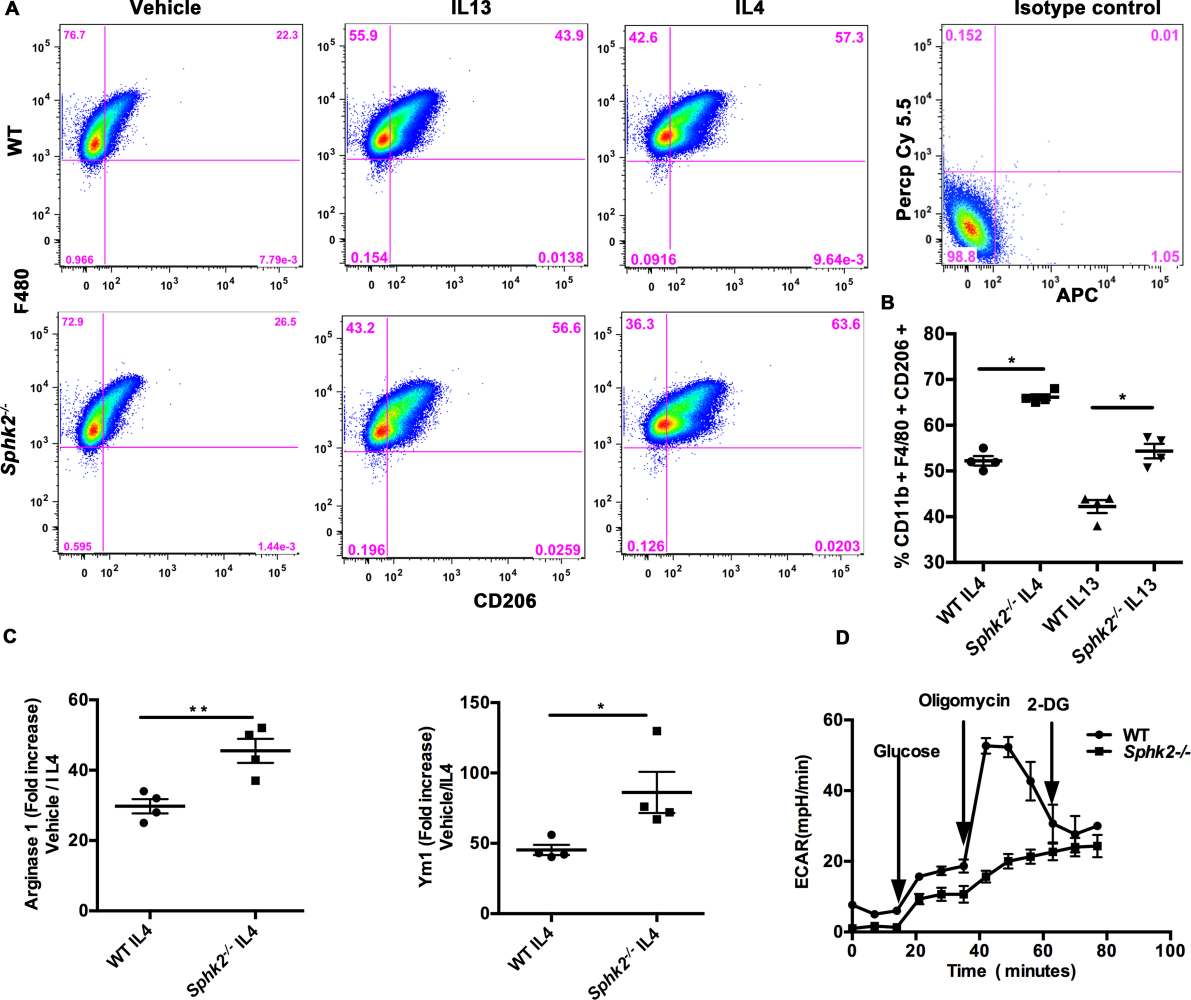
Bone marrow derived macrophages from *Sphk2*^*-/-*^ mice preferentially polarize towards the M2 phenotype in the presence of M2 promoting signals. Bone marrow cells isolated from WT and *Sphk2*^*-/-*^ mice were differentiated into macrophages in medium containing Macrophage Colony stimulating growth factor (MCSF) and stimulated either with IL4 or IL13 for 24 hours. Flow cytometry analysis shows that *Sphk2*^*-/-*^ mice were able to polarize significantly (p<0.05) towards M2 macrophages positive for CD11b^+^ F4/80^+^ CD206^+^. Pseudo color plot (A) and percent over control increase (B) for CD206^+^ cells is shown. QRT PCR analysis on total RNA prepared from IL4 stimulated macrophages from WT and *Sphk2*^*-/-*^ macrophages revealed that mRNA expression levels for M2 markers Arg-1 and Ym-1 (C) were increased in BMDMs from *Sphk2*^*-/-*^ mice compared to WT cells. (Data represents average of 3 experiments, n = 3/experiment). (D) Glycolytic rate in BMDM as assessed by the extracellular acidification rate (ECAR) using the Seahorse XF analyzer showed a significant increase in the glycolytic rate which was further enhanced upon addition of the glycolytic driver Oligomycin in WT but not *Sphk2*^*-/-/*^ cells, indicating that the glycolytic pathway is largely inactive in *Sphk2*^*-/-*^ cells. Suppression of glycolysis with 2-DG has no effect on *Sphk2*^*-/-*^ cells. **p<0.01; *p<0.05. Data represents average of 2 experiments, experiments repeated at least two times with N = 6 each time.

To determine if the lack of Sphk2 predisposes a cell-intrinsic M2 polarization, we cultured bone marrow derived macrophages (BMDM) from WT and *Sphk2*^*-/-*^ mice in the presence of MCSF and M2-promoting IL-4 or IL-13 cytokines. Phenotypic analysis of cells by flow cytometry after 24 hours of cytokine stimulation indicated increased percentages of M2 macrophages (CD11b^+^, F4/80^+^, CD206^+^) in the *Sphk2*^*-/-*^ cultures compared to WT under identical cytokine concentrations (Fig 5A and 5B), which was accompanied by increase in mRNA levels of M2 markers Arg-1 and Ym-1 (Fig 5C). These results suggest that deficiency of Sphk2 promotes the pro-healing M2 macrophage differentiation program, contributing to attenuated renal damage in response to ureteral obstruction.

Pro-inflammatory M1 macrophages undergo a metabolic switch toward increased glycolysis and reduced oxidative phosphorylation [18]. Altered metabolism is not only a characteristic of polarized macrophage subsets but is also a prerequisite for proper polarization and inflammatory regulation. Inhibition of glycolysis or oxidative phosphorylation has been demonstrated to impair M1 or M2 activation respectively [25], suggesting that the macrophage metabolic profile is indicative of polarization status and inflammatory potential. We determined the glycolytic rate of WT and *Sphk2*^*-/-*^ BMDMs by assessing the extracellular acidification rate (ECAR) using the Seahorse XF analyzer [18]. The addition of glucose significantly increased the glycolytic rate in WT BMDM, which was further enhanced upon addition of the glycolytic driver Oligomycin and strongly decreased upon inhibition of glucose hexokinase with 2 deoxyglucose (2DG). However, this pattern was not evident in *Sphk2*^*-/-*^ cells indicating that the glycolytic pathway is largely inactive in the absence of Sphk2 (Fig 5D). Therefore, these results imply that the increase in M2 macrophages in *Sphk2*^*-/-*^ mice may be due to impaired macrophage metabolism and that Sphk2 may regulate the glycolytic pathway.

### An Sphk2 inhibitor diminishes renal injury

**Fig 6:**
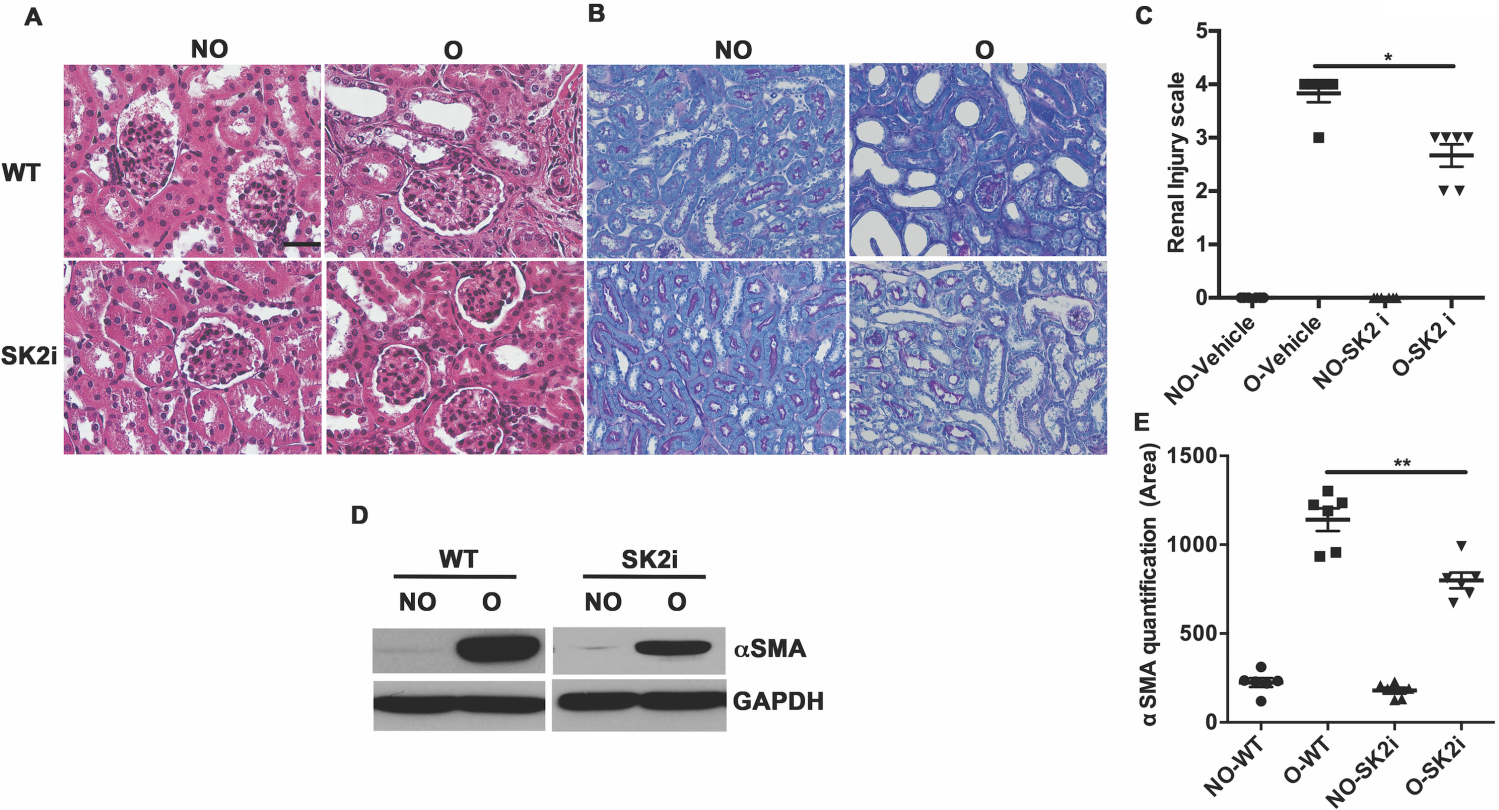
Sphk2 inhibitor treatment diminishes renal injury. 6–8 week old WT mice were treated with the Sphk2 inhibitor (SK2i) at 3mg/kg. Renal injury was assessed in H&E stain (A), PAS stain (B) and scored (C). Myofibroblast infiltration indicated by protein levels of **α**SMA is significantly lower in SK2i treated mice (D), protein bands quantified using Image J and normalized to GAPDH (E). **p<0.01; *p<0.05. N = 6. Experiments repeated at least two times with N = 6 each time. Original magnification; 20x objective. Scale bar; 50μm.

**Fig 7:**
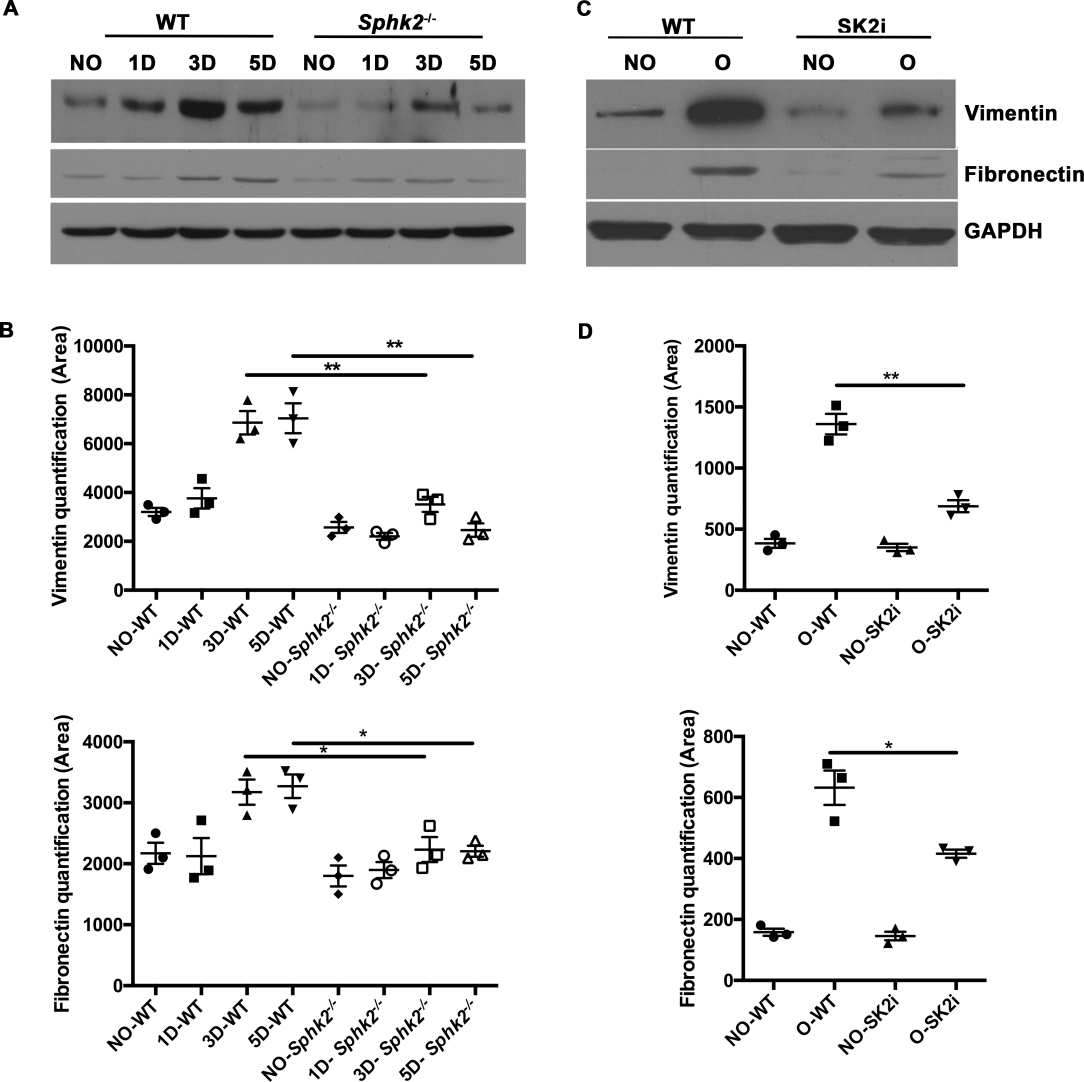
Reduced EMT transition in absence of Sphk2 following renal injury. 6–8 week old WT, Sphk2^-/-^ or WT mice were treated with Sphk2 inhibitor (SK2i) at 3mg/kg or vehicle control, daily by i.p three days prior to and following the UUO surgery (6 days total) and expression of EMT markers were assessed in whole kidney lysates. Western Blot analysis indicated reduced expression of EMT markers Vimentin and Fibronectin in Sphk2^-/-^ (A, B) and SK2i treated mice (C, D), protein bands were quantified using Image J and normalized to GAPDH. **p<0.01; *p<0.05. N = 3.

**Fig 8:**
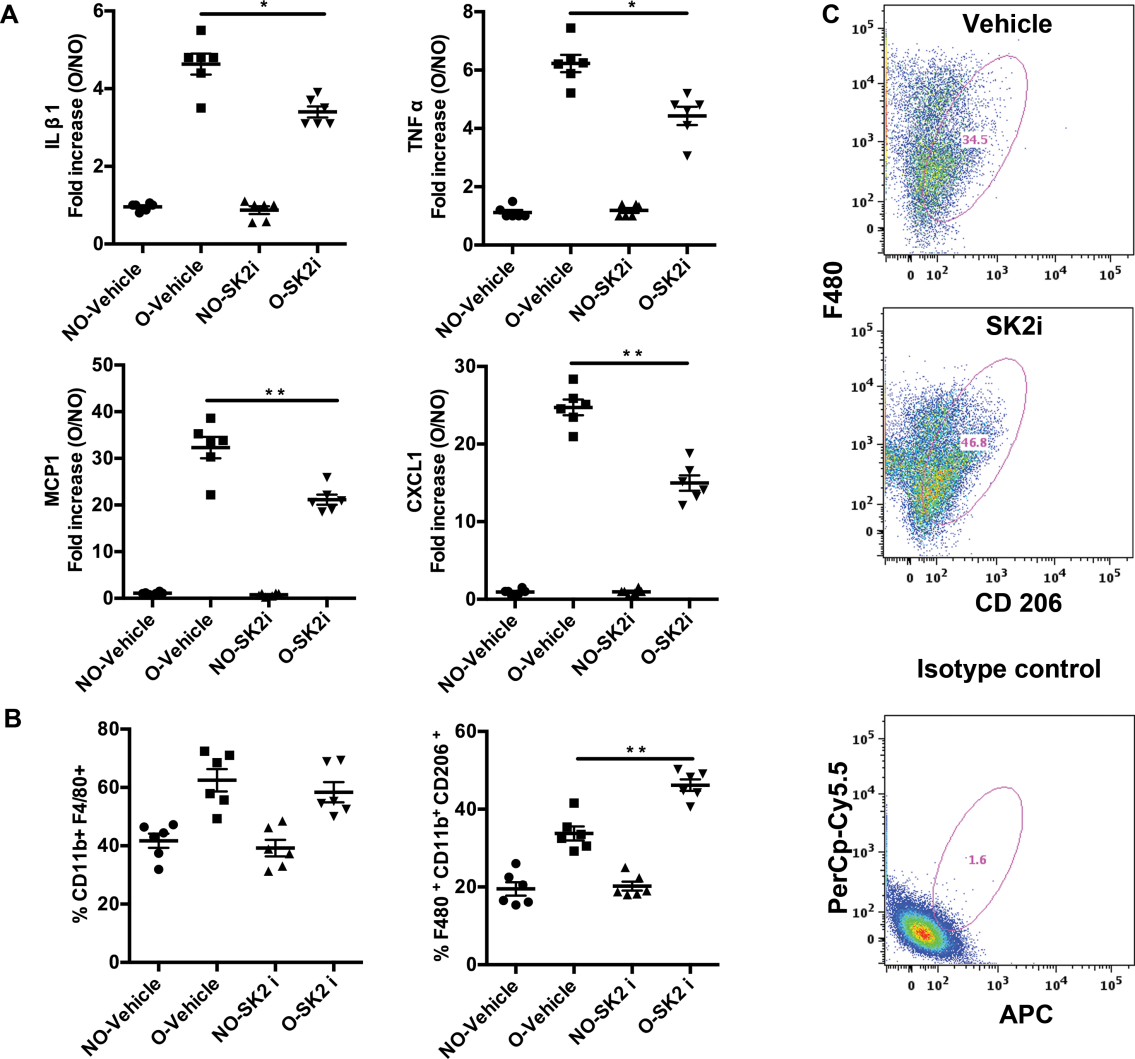
Inflammatory cytokines are diminished in mice treated with SK2i. Mice subjected to UUO were treated with either vehicle (2%cyclodextrin) or SK2i (3mg/kg) and kidney tissue was harvested for qRT PCR analysis for inflammatory cytokine mRNA shows that expression levels of IL1**β**, TNF**α**, MCP-1 and CXCL1 were significantly lower in SK2i compared to vehicle treated group, N = 6. (B) Flow Cytometry analysis of isolated kidney cells show that the percentage CD11b^+^F4/80^+^ cells did not change in vehicle and SK2i treated mice whereas the proportion of anti-inflammatory M2 cells (F4/80^+^ CD11b^+^ CD206^+^) is significantly higher (C) in the SK2i group. Fig 7C shows pseudo color plots for CD206^+^ cells of vehicle and SK2i treated mice and isotype control. (**p<0.01; *p<0.05). (Data represents average of 3 experiments, n = 3/experiment).

While genetic ablation is invaluable in analyzing the contribution of a particular gene to a biological process, the ultimate goal is to reproduce the favorable outcome pharmacologically. We obtained a Sphk2 inhibitor SLP120701 (SK2i) with characterized potency and specificity developed by SphynKx Therapeutics [3,7,26]. Administration of the SK2i to WT mice resulted in diminished Sphk2 protein expression 5 days post obstruction (S2A Fig) along with a substantial increase in plasma S1P levels that mimicked the phenotype of *Sphk2*^*-/-*^ mice, indicative of a systemic effect (S2B Fig) and recapitulating the Sphk1-dependent elevation of circulating S1P levels as previously published [3]. Importantly, treatment of *Sphk2*^*-/-*^ mice with the SK2i showed no effect on plasma S1P levels, verifying that the inhibitor specifically targets Sphk2. Renal histopathology was evaluated in mice treated with the inhibitor or vehicle controls at 3 days post UUO surgery. Similar to results obtained from the *Sphk2*^*-/-*^ mice, SK2i treated mice showed improved histology with diminished renal injury, minimal inflammatory interstitial infiltrates, cortical thickening and tubular dilatation and intact proximal tubule brush borders (Fig 6A–6C). Further investigation demonstrated that protein levels of **α**SMA (Fig 6D and 6E), Vimentin and Fibronectin (Fig 7) were significantly lower in the Sphk2^-/-^ and SK2i treated group while the overall increase in mRNA expression of the proinflammatory cytokines IL-**β**1, MCP-1, TNF**α** and CXCL1 following obstruction was significantly attenuated by SK2i treatment (Fig 8A). Finally, a higher percentage of the total kidney cells isolated from SK2i-treated mice at 3 days post-surgery were macrophages (CD11b^+^ F4/80^+^, Fig 7B and 7C) and a higher percentage of these displayed the pro-healing M2 phenotype (F4/80^+^ CD11b^+^ CD206^+^, Fig 8B and 8C). Therefore, specific inhibition of Sphk2 by this SK2i recapitulates the beneficial effects of *Sphk2* deletion, supporting Sphk2 as a viable therapeutic target for mitigating renal injury.

### Renal fibrosis is diminished in SK2i treated mice

**Fig 9:**
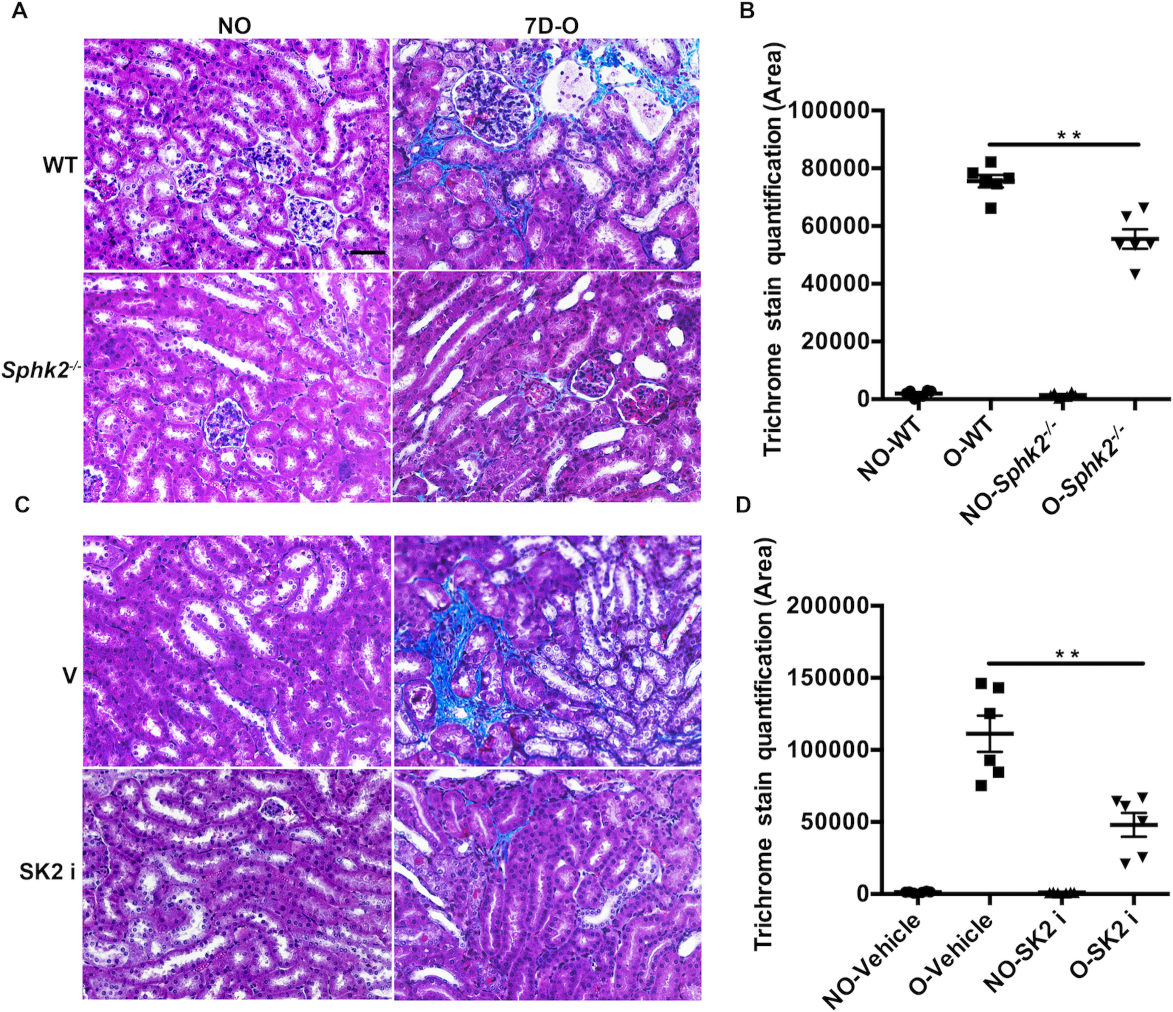
Diminished renal fibrosis in *Sphk2*^*-/-*^ and SK2i treated mice. *Sphk2*^*-/-*^ mice and WT treated with SK2i were subjected to UUO for 7 days with appropriate control groups. Masson’s Trichrome staining indicates reduced renal fibrosis after 7 days of obstruction in *Sphk2*^*-/-*^ (A&B) and SK2i treated mice (C&D) compared non-obstructed controls and quantitated by Image J. N = 6. (Data represents average of 3 experiments, n = 3/experiment). **p<0.01). Original magnification; 20x objective. Scale bar; 50μm.

Renal damage from injury and inflammation is often followed by irreparable scarring and fibrosis resulting in permanently compromised renal function. To determine if the improved structural and inflammatory outcomes observed at the early 3-day, pro-inflammatory phase translates to improved renal health long term, we evaluated renal fibrosis in mice at the fibrotic phase 7 days post-obstruction. Indeed, trichrome staining of kidney sections indicated increased levels of collagen deposition (indicative of fibrosis, blue color) in WT as compared to *Sphk2*^*-/-*^ mice (Fig 9A and 9B). Similarly, treatment with the SK2i significantly decreased the fibrotic area in WT kidneys as compared to vehicle treated samples at 7 days post obstruction (Fig 9C and 9D). Therefore, the protective effects of Sphk2 deficiency at early stages of injury serve to reduce fibrosis long term and preserve renal integrity.

### Both hematopoietic cells and the kidney parenchyma contribute to protection due to *Sphk2* deletion

**Fig 10:**
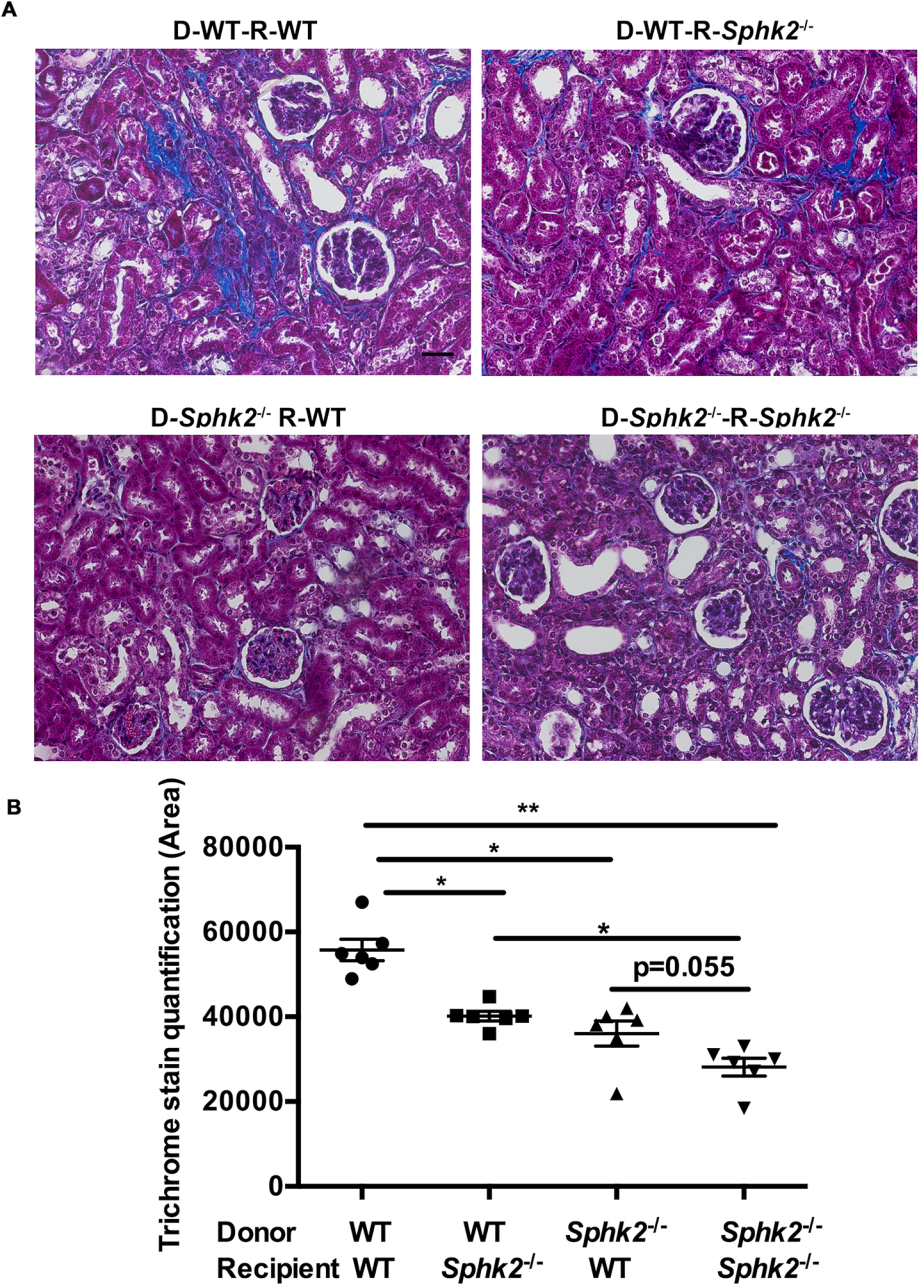
Sphk2 in both immune cells and kidney microenvironment contribute to renal fibrosis. (A). Recipient wild-type and *Sphk2*^-/-^ mice were irradiated and injected with 1 × 10^6^ bone marrow cells obtained from WT or *Sphk2*^-/-^ mice by tail vein. 8 weeks after engraftment mice were subjected to UUO for 7 days and trichrome staining performed on kidney sections. Image J quantification (B) indicates that WT mice receiving WT cells have the highest degree of renal fibrosis, followed by *Sphk2*^*-/-*^ recipients receiving WT cells and WT mice receiving *Sphk2*^*-/-*^ bone marrow. *Sphk2*^*-/-*^ mice receiving *Sphk2*^*-/-*^ transplanted cells had the least fibrosis. N = 4/genotype. **p<0.01; *p<0.05. Original magnification; 20x objective. Scale bar; 50μm.

While our earlier *in vitro* studies demonstrating the pro-M2 bias of isolated *Sphk2*^*-/-*^ macrophages suggest that this effect is cell intrinsic, it is possible that the Sphk2 expressed in the kidney parenchyma also influences renal damage. To directly address this possibility, we performed bone marrow transplant of irradiated recipient WT and *Sphk2*^-/-^ mice with bone marrow from either WT or *Sphk2*^-/-^ mice. Transplant efficiency and consistency among groups was confirmed by analysis of circulating leukocyte subsets repopulating the peripheral blood by flow cytometry after 6 weeks (S3B Fig); the ureters of chimeric mice obstructed eight weeks after engraftment and kidneys were harvested 7 days post-obstruction. Trichrome staining of the renal tissue indicated that the fibrotic area was maximal in wild type mice receiving wild type cells (both kidney parenchyma and hematopoietic cells are WT) and lowest in *Sphk2*^*-/-*^ mice receiving *Sphk2*^*-/-*^ transplants (both parenchymal and hematopoietic cells *Sphk2*^*-/-*^, Fig 10A and 10B). Importantly, fibrosis in WT mice receiving *Sphk2*^*-/-*^ bone marrow (kidney parenchyma WT and *Sphk2*^*-/-*^ hematopoietic cells) and the reverse combination, *Sphk2*^*-/-*^ mice receiving WT bone marrow cells (kidney parenchyma *Sphk2*^*-/-*^ and WT hematopoietic cells) were both significantly lower than WT mice receiving WT cells. Taken together, these results suggest that Sphk2 expression on the kidney parenchymal as well as the hematopoietic cells both contribute to drive the pro-inflammatory phenotype in WT mice, but the lack of Sphk2 expression in either tissue is sufficient to significantly reduce renal fibrosis.

## Discussion

To date, Sphk1 has been the most actively investigated of the two sphingosine kinases and has generally been associated with pro-survival and anti-apoptotic functions. In studies manipulating levels in vivo, Sphk1 was shown to be renoprotective, primarily through its generation of S1P and subsequent activation of one of its five receptors, S1PR1 [27,28]. Paradoxically, SphK1 is upregulated in a variety of inflammatory kidney diseases [5,29], but it is believed that increased inflammatory Sphk1 levels reflect a compensatory response to damage rather than a pro-inflammatory role for the enzyme. Alternatively, the functional effects of Sphk2 appear to be essentially the opposite of those attributed to Sphk1, as illustrated by studies wherein the genetic absence of Sphk2 is protective in ischemia-reperfusion injury of the kidney [30] and liver [31] by diminishing cell cycle arrest and apoptosis [4]. In addition, pharmacological inhibition of Sphk2 results in anti-inflammatory effects in a number of murine disease models including ulcerative colitis [32], Crohn’s disease [33] and inflammatory arthritis [34], implying that reduced Sphk2 activity attenuates inflammation and injury in agreement with our findings.

Importantly, while the majority of studies support a role for Sphk2 as a pro-inflammatory molecule, the genetic ablation of *Sphk2* also triggers a compensatory increase in the activity of Sphk1 and subsequent elevation of circulating S1P levels, raising the real possibility that the beneficial effects of Sphk2 inhibition are largely due to amplified S1P activity [35]. Confounding these findings is the fact that in arthritis models, genetic deletion of Sphk2 did not recapitulate the beneficial effects achieved by pharmacologic inhibition [34,36,37]. This discrepancy might suggest that the inhibitor used in these studies affects other molecules or pathways. Alternatively, this may indicate that that the Sphk2 protein has additional functions that are independent of its kinase activity and are lost only when the protein is completely absent. The current study is an effort to clarify these inconsistencies and further explore the potential of Sphk2 as a therapeutic target in treatment of renal injury.

Our current study proposes Sphk2 as a catalyst of injury-induced inflammation, as both its inhibition and genetic ablation clearly reduces key aspects of inflammation in response to UUO, including expression of critical pro-inflammatory and fibrogenic cytokines [38]. Because cytokines produced by infiltrating inflammatory macrophages contribute to ongoing inflammation and the progression of fibrosis, it is possible that the overall dampening of pro-inflammatory signals in the kidney environment in the absence of Sphk2 activity may underlie the diminished fibrosis. Similarly, the effective rescue of the protective phenotype by *Sphk2*^-/-^ bone marrow in WT mice argues that the infiltrating hematopoietic cells contribute significantly to pro-healing phenotype, in agreement with published studies demonstrating a universal role for bone marrow-derived cells in promoting kidney fibrosis [39]. Alternatively, we show a higher proportion of M2 macrophages in obstructed kidneys of *Sphk2*^*-/-*^ mice and propose that this M2-bias also promotes the protective outcome. While it is clear that dysregulation of either arm of the healing process generates unremitting inflammation and/or maladaptive repair leading to tissue-destructive fibrosis, studies in which the balance of macrophage populations was adjusted to favor the reparative pro-healing M2 phenotype largely report improved tissue repair [40–52]. However, other studies imply that sustained activation or prolonged recruitment of M2 populations may also contribute to pathological fibrosis by producing important pro-fibrotic mediators such as TGF-**β**1 [53]. In the kidney, deletion or inhibition of various M1 stimuli in UUO and other renal injury models lead to an increase in M2 macrophages and generally reduced destruction and fibrosis [40–49], in agreement with our results. Similarly, an M2-bias lead to improvement [50–52] or impairment [54] of fibrosis in coronary injury models, while exacerbated renal [55] and pulmonary fibrosis [56,57] in the presence of an M2-bias has been reported; clearly these processes are mediated by mechanisms far more complex than the balance of macrophage subsets [58,59]. Future studies focusing specifically on questions such as differential or tissue-specific recruitment of various and additional novel macrophage subsets will undoubtedly clarify these issues.

The mechanisms guiding the ultimate phenotype of infiltrating macrophages, the rules that orchestrate the various interrelated phases or the complete spectrum of macrophage subsets remain unclear [58–61]. Our findings are consistent with observations that S1P itself can stimulate a significant M2 polarization via S1P-induced secretion of the M2-promoting cytokines IL-4 and IL-13 [62,63]. Alternatively, an S1P-dependent macrophage polarization toward the M1 phenotype in an Sphk2-deficient tumor xenograft model has been reported [64]. These paradoxical findings regarding S1P-dependent polarization likely reflect differences in the cytokines induced by S1P in the tumor microenvironment vs. isolated macrophages, but raise the possibility that the elevated plasma S1P levels in *Sphk2*^*-/-*^ mice are responsible for the differential polarization of macrophages in the kidney in our study. However, we observe a similarly skewed M2 polarization of *Sphk2*^*-/-*^ macrophages *in vitro* even in the presence of high concentrations of IL-4 and IL-13, suggesting our phenotype does not rely on signaling via S1P to produce these M2 cytokines, and thus is S1P-independent. Recently, immunometabolism, or the metabolic reprogramming of cells in response to immune stimuli, has been shown to link receptor-mediated immune signal transduction to metabolic pathways that determine the lineage fate and support the unique functions of immune cell subsets (reviewed in refs [65–68]). Rapidly activated, highly proliferative cells such as pro-inflammatory M1 macrophages and effector T cells rely on glycolytic, amino acid metabolic, fatty acid synthetic and the pentose phosphate pathways to produce the energy and synthetic intermediates necessary for their stimulatory functions. Alternatively, tolerogenic/anti-inflammatory immune signals stimulate fatty acid oxidation, the TCA cycle and the arginase pathway to support functions of the M2 macrophages, Treg cells and memory CD8+ cells [68]. Inhibition or dysregulation of many of the steps of these pathways or subsequent regulatory pathways impairs polarization [40,44,50,69,70]. We show that Sphk2 is a novel regulator of macrophage polarization and that *Sphk2*^*-/-*^ macrophages have severely impaired glycolytic activity, which might be responsible for the M2 bias. Furthermore, this intrinsic bias supports a role for Sphk2 as a fundamental inflammatory regulator that specifies the pro-inflammatory macrophage gene expression program and a potential target for ameliorating inflammatory fibrosis of any cause. Investigation into the novel or existing metabolic processes regulated by Sphk2, the mechanisms of this regulation and the potential to identify novel therapeutic immunometabolic targets is our current research focus.

Our results from bone marrow transplant experiments support a role for the kidney parenchyma in the Sphk2-mediated inflammatory response. Interestingly, in support of this data and a metabolic basis for Sphk2-mediated renal injury, investigations using the IRI model of CKD demonstrated that proximal tubule epithelial cells also undergo a metabolic switch to glycolysis early after injury that eventually reverts to allow recovery in normal tubular cells [71,72]. However, a percentage of cells fail to undergo the metabolic reversal, becoming progressively more atrophic with increased mitochondrial loss, expression of glycolytic enzymes and other indicators of unchecked glycolysis. Furthermore, therapeutic reversal of fibrosis has been attributed to inhibition of metabolic pathways, including lipid metabolism and glycolysis [73]. When taken in the context of our results regarding the decreased glycolytic potential in Sphk2-null macrophages, it is logical to postulate this phenotype extends to the proximal tubule cells. In this case, Sphk2 expression would trigger and sustain the glycolytic switch in tubular epithelium as well, promoting an adverse metabolic fate, development of atrophy and increased fibrosis contributing to the renal pathology following renal injury. Experiments are underway to investigate this intriguing possibility.

Finally, two recent studies investigated Sphk2 in renal injury by manipulating Sphk2 levels or activity in the UUO model [9] or chemical and ischemic renal injury models [8] whose results strongly support our findings that Sphk2 exacerbates renal fibrosis. While these studies had a similar focus, they each assessed response to injury at different time points post injury (3 days (this study), 7d [9] and 14d [8]) which logically would result in different conclusions regarding underlying mechanism, since the healing process includes the highly temporally orchestrated regulation of inflammation, fibrosis and resolution [48,74–76]. In the current study we investigated injury at 3d, the early, pro-inflammatory phase of the response that is primarily mediated by infiltrating monocytes and pro-inflammatory M1 macrophages that function to clear cell debris and call in more inflammatory cells in preparation for the M2 or pro-healing phase at 5d. We determined that at 3d, macrophage profiles in the Sphk2^-/-^ kidneys are skewed toward the pro-healing M2 population, suggesting a role for Sphk2 in macrophage polarization to promote inflammation and fibrosis. In agreement with our findings, Schwalm et al. [9] observed reductions in inflammation, pro-fibrotic signaling and fibrosis at the fibrotic phase, 7 days post-obstruction, upon Sphk2 deletion. Increased tissue sphingosine levels in the absence of Sphk2 led to the hypothesis that the lack of Sphk2 caused a buildup of its substrate sphingosine, leading to SMAD7 activation, decreased activation of the TGF**β**/Smad2/Smad3 pathway and subsequent reduced expression of the fibrotic proteins that are critical to fibrotic repair at this phase of inflammation [9]. Finally, Bajwa et al assessed the late, 14d post-injury response and determined that the protective effects of Sphk2 loss persisted during this longer-term progression to chronic kidney disease. These investigators correlated decreased fibrosis in the absence of Sphk2 with increases in CD4^+^ T cell-derived IFN-γ and the IFN-γ-responsive chemokines Cxcl9 and Cxcl10. Their findings associating T cells with reduced fibrosis are in accord with deployment of the adaptive immune response following the inflammatory and fibrotic phases. Paradoxically, IFN-γ and related factors are generally considered to be pro-inflammatory, pro-fibrotic and toxic when present at early time points (reviewed in ref [77]) where IFN-γ promotes the prototypic M1, pro-inflammatory phenotype [59], suggesting that the high IFN-γ levels at later time points serve to decrease inflammation and fibrosis. Therefore, while these studies appear to be similar on the surface, their focus on very different responses further supports the utility of Sphk2 as a therapeutic target in multiple phases during the response to sterile injury.

Collectively, we show that results in genetically deficient animals are recapitulated by a novel pharmacologic inhibitor, thus implicating Sphk2 as a viable target in ameliorating obstruction-induced renal injury. By preferentially polarizing reparative M2 macrophages, lack or inhibition of Sphk2 results in diminished expression of inflammatory cytokines and reduced fibrosis at the site of injury. While aspects of the S1P signaling pathway have been well studied in the setting of renal injury, our findings, particularly as they relate to macrophage polarization, suggest that Sphk2 may play a role independent of S1P in determining macrophage phenotypic fate. These findings may have applicability to pathologies in numerous tissues that involve wound repair in response to tissue damage or injury. Moreover, further investigation into potential alternate Sphk2-dependent pathways or substrates will provide valuable clues to mechanisms responsible for immune cell-mediated regulation of inflammation and healing. Finally, these studies support the further investigation of Sphk2 inhibitors as novel therapies for modulating inflammatory injury and potentially, chronic inflammatory conditions.

## Disclosures

All animal experiments in this study were reviewed and approved by Animal Care Committee at UCONN Health. All the authors do not have any conflict of interest.

## Supporting information

S1 Fig **(A) Basal immune profile of bone marrow cells in WT and *Sphk2***^***-/-***^ **mice.** Flow cytometry studies revealed that basal immune cell profiles of peripheral blood, bone marrow, kidney, spleen and lymph nodes were similar between the genotypes. We have shown immune profile of bone marrow cells in the figure. **(B) Sphk2 protein expression is upregulated following renal injury**. 6–8 week old WT mice were subjected to UUO and Sphk2 protein levels were assessed in kidney lysates over time.
(TIF)

S2 Fig SK2i treatment reduces Sphk2 protein expression with elevated plasma S1P levels and reduces renal injury in WT mice. (A). Diminished renal expression of Sphk2 in WT mice treated with SK2i (3mg/kg) following UUO. (B). Circulating S1P levels as analyzed by Liquid Chromatography- ESI Mass Spectrometry (LC-MS), were significantly increased in SK2i treated mice and Sphk2^-/-^ mice compared to WT and vehicle treated group, n = 6.
(TIF)

S3 Fig Pseudo-color plots of gating strategy used in kidney cells for in vivo studies or bone marrow derived macrophages for in vitro studies. (A). Figure shows sequential gating to obtain a CD45^+^ hematopoietic cell population. T and B Lymphocytes, Ly6G^+^ neutrophils and natural killer cells were gated out of the live cell population and the remaining CD45^+^ cells were analyzed for CD11b^+^ F4/80^+^ CD206^+^.
(**B**). Flow cytometry analysis of leukocytes obtained from peripheral blood of indicated groups of mice 6 weeks post-transplant. %CD45 cells confirmed transplant efficiency and consistency among experimental groups.
(TIF)
