# Supplementary figures and images for: Cell-intrinsic sphingosine kinase 2 promotes macrophage polarization and renal inflammation in response to unilateral ureteral obstruction
Source: PLoS One. 2018 Mar 8;13(3):e0194053. doi: 10.1371/journal.pone.0194053 (PMC5843290; doi:10.1371/journal.pone.0194053)

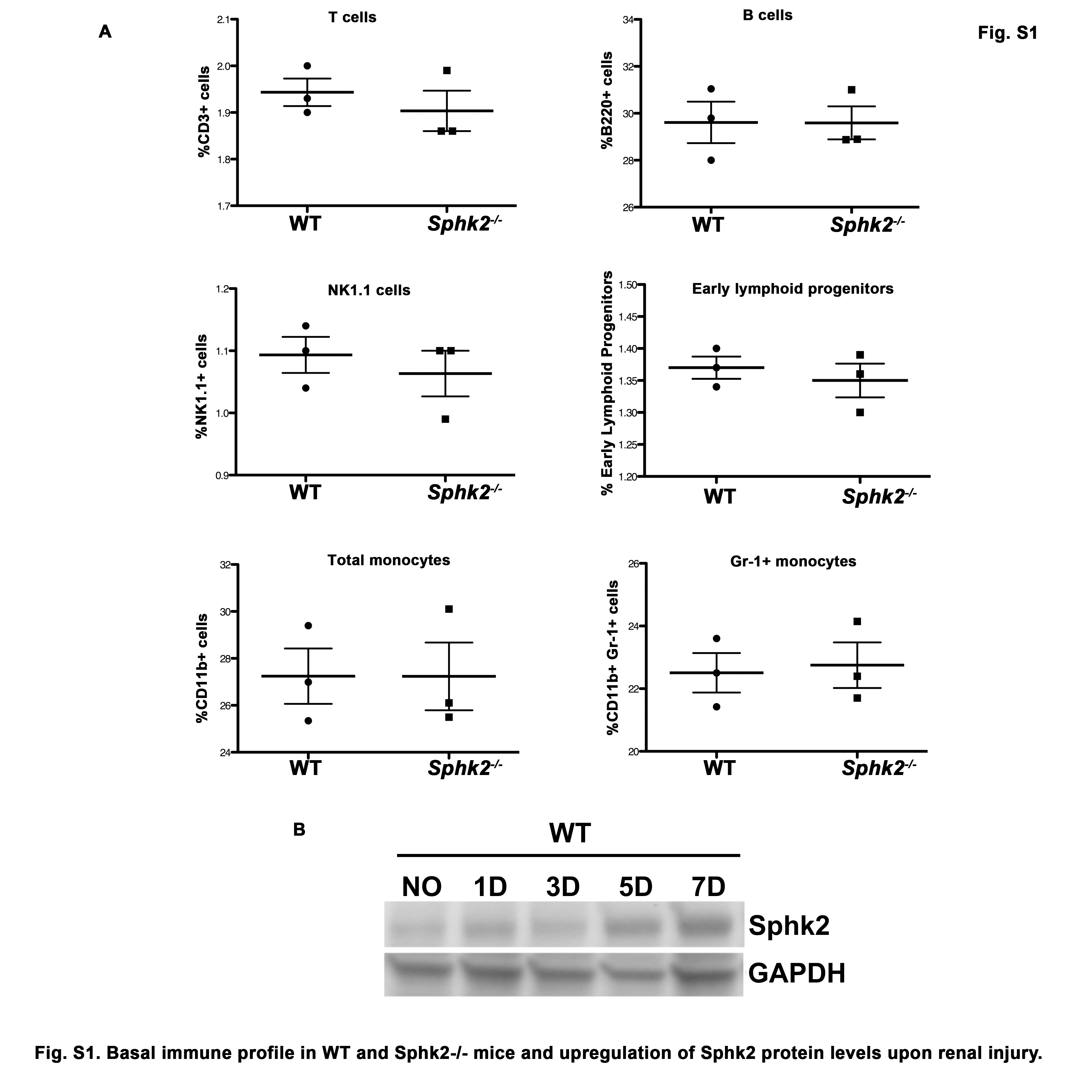

Supplement: S1 Fig — (A) Basal immune profile of bone marrow cells in WT and Sphk2-/- mice. Flow cytometry studies revealed that basal immune cell profiles of peripheral blood, bone marrow, kidney, spleen and lymph nodes were similar between the genotypes. We have shown immune profile of bone marrow cells in the figure. (B) Sphk2 protein expression is upregulated following renal injury. 6–8 week old WT mice were subjected to UUO and Sphk2 protein levels were assessed in kidney lysates over time. (TIF) [file pone.0194053.s001.tif]

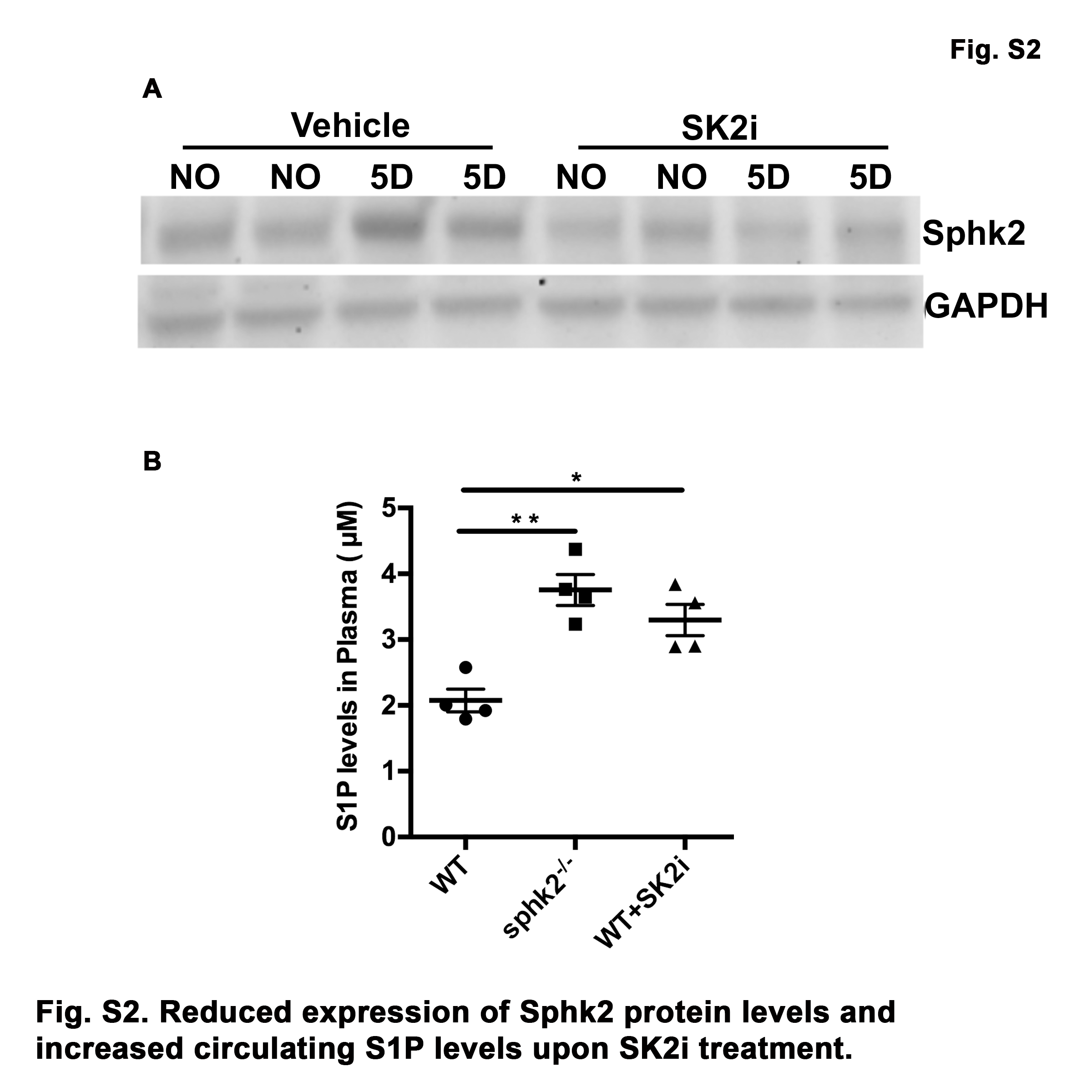

Supplement: S2 Fig — (A). Diminished renal expression of Sphk2 in WT mice treated with SK2i (3mg/kg) following UUO. (B). Circulating S1P levels as analyzed by Liquid Chromatography- ESI Mass Spectrometry (LC-MS), were significantly increased in SK2i treated mice and Sphk2-/- mice compared to WT and vehicle treated group, n = 6. (TIF) [file pone.0194053.s002.tif]

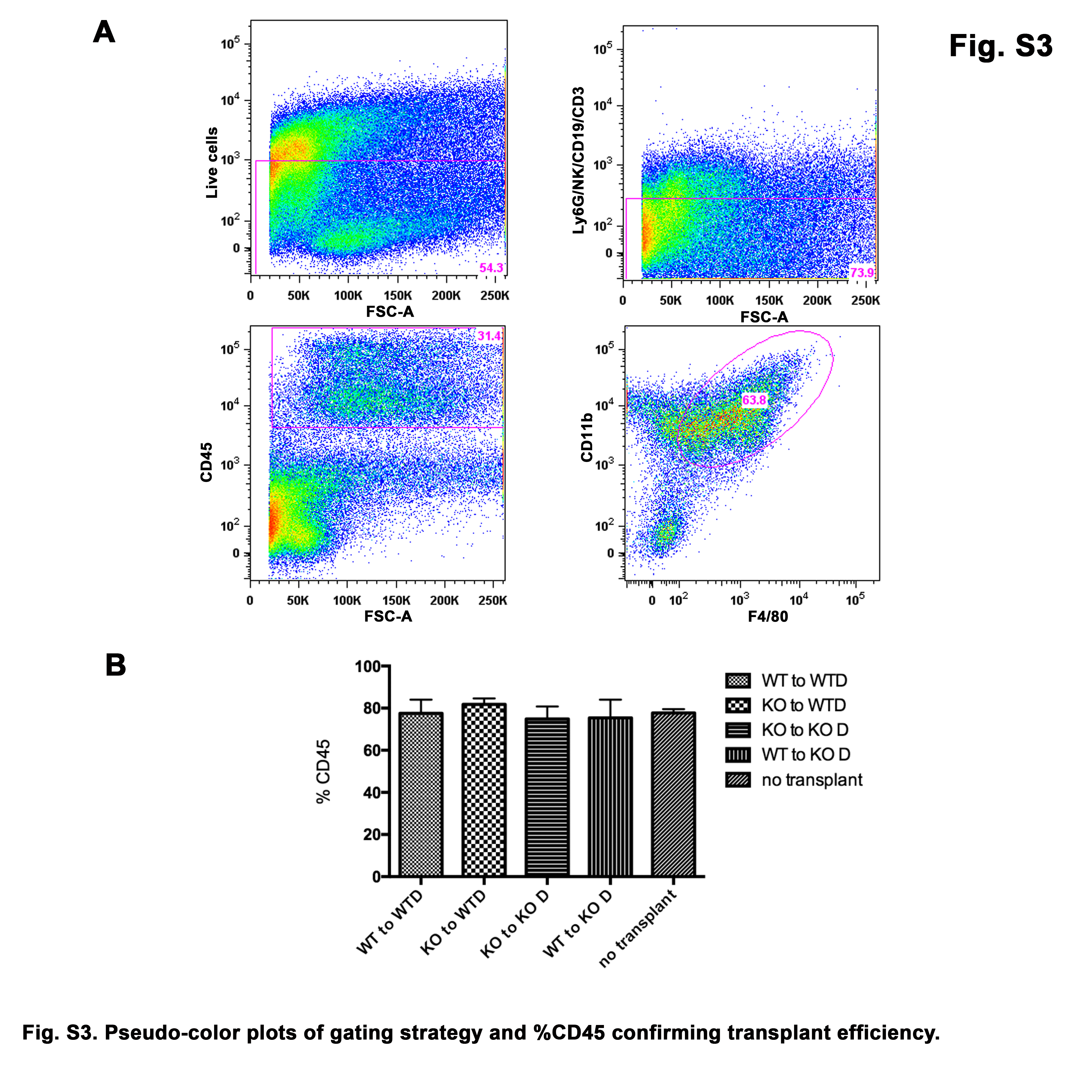

Supplement: S3 Fig — (A). Figure shows sequential gating to obtain a CD45+ hematopoietic cell population. T and B Lymphocytes, Ly6G+ neutrophils and natural killer cells were gated out of the live cell population and the remaining CD45+ cells were analyzed for CD11b+ F4/80+ CD206+. (B). Flow cytometry analysis of leukocytes obtained from peripheral blood of indicated groups of mice 6 weeks post-transplant. %CD45 cells confirmed transplant efficiency and consistency among experimental groups. (TIF) [file pone.0194053.s003.tif]
